# Supplementary material for: Identification of Efflux Pump Mutations in Pseudomonas aeruginosa from Clinical Samples
Source: Antibiotics (Basel). 2023 Mar 1;12(3):486. doi: 10.3390/antibiotics12030486 (PMC10044530; doi:10.3390/antibiotics12030486)
Supplement: Supplementary file 1 [file antibiotics-12-00486-s001.zip › antibiotics-2199385-supplementary.pdf]

Supplementary Files

Identification of Efflux Pump Responsible for Antibiotic Resistance in *Pseudomonas aeruginosa* from Clinical Samples and In Silico Drug Designing

Sonia Quddus[1], Zainab Liaqat[1], Sadiq Azam[1], Mahboob ul Haq[2], Sajjad Ahmad[3*], Metab Alharbi[4], Ibrar Khan[1*]

[1]Centre of Biotechnology and Microbiology, University of Peshawar, Pakistan
[2]Department of Pharmacy, Abasyn University, Peshawar 25000, Pakistan.

[3]Departments of Computer Science and Physics, Center for Soft Matter and Biological Physics, Virginia Tech, Blacksburg, Virginia 24060, USA.

[4]Department of Pharmacology and Toxicology, College of Pharmacy, King Saud University, P.O.Box 2455, Riyadh 11451, Saudi Arabia.

Correspondence

Sajjad Ahmad and Ibrar Khan

Table S1. Dynamut result of mexA and mexB.

Gene	delta_vibrational_entropy	ddg_prediction	delta_stability_encom	Mcsm	mutation	sdm	duet
MEX A	0.065	0.075	-0.052	0.032	E178K	0	0.363
	0.503	-0.242	-0.403	-0.371	S32P	-1.18	-0.479
	0.521	-0.11	-0.417	-0.31	K54G	0.74	0.059
	0.177	-0.377	-0.142	-0.301	T55V	0.83	0.132
	-0.141	0.255	0.113	-0.003	T59D	-0.36	0.183
	0.026	0.15	-0.021	-1.091	A110Y	0	-0.933
	-0.053	0.38	0.042	-0.377	S33Q	0.28	0.006
	0.04	-0.415	-0.032	-0.773	G44K	-2.62	-1.043
	0.571	-0.788	-0.457	-0.561	K69N	-0.91	-0.44
	-0.353	0.123	0.282	-0.454	G100I	-2.36	-0.817
	-0.06	-0.569	0.048	-0.146	I94Q	-1.29	-0.03
	0.488	-1.075	-0.39	-1.583	L57S	-2.49	-1.76
	0.017	0.372	-0.013	-0.013	N71I	1.51	0.559
MEX B	-0.036	0.279	0.029	-0.579	N28T	-0.27	-0.401
	0.427	-0.597	-0.342	-1.31	W4T	-0.28	-1.084
	-1.542	1.62	1.233	-1.691	A83K	-3.23	-1.906
	-0.027	0.6	0.021	0.134	Q30L	1.19	0.644
	0.013	-0.447	-0.01	-0.311	G74N	-1.44	-0.369
	-0.059	-0.001	0.047	-0.537	P40I	-0.03	-0.218
	-0.33	0.365	0.264	-0.849	P7F	0.98	-0.381
	0.032	0.781	-0.026	-0.978	S101W	1.5	-0.331
	-0.131	0.656	0.105	0.67	Q62M	0.54	0.971
	0.074	-0.146	-0.059	-0.233	Q58T	0.34	0.167
	-0.088	-0.031	0.07	-0.264	N43T	0.2	0.039
	0.116	-0.944	-0.093	-1.066	K80S	-1.6	-1.179
	0.893	0.157	-0.714	-0.742	R78P	-1.91	-1.187

-0.137	0.037	0.11	-0.064	Q97S	-0.91	0.155
0.003	-0.01	-0.002	-0.294	S22N	0.42	0.162
-0.109	1.418	0.087	-0.239	S75I	2.08	0.451
1.336	-2.441	-1.069	-2.853	L68S	-3.89	-3.204
0	0.273	0	0.431	Y106K	-0.03	0.715
-0.31	0.557	0.248	-0.418	D6T	-0.22	-0.246
0.934	-0.319	-0.747	-0.79	Y92G	0.3	-0.514
-0.115	0.232	0.092	-0.281	Q109S	-0.57	-0.087
-0.022	-0.005	0.018	0.38	H46V	0.01	0.425
-0.278	0.821	0.223	-0.198	T50L	0.99	0.232
-0.059	-0.313	0.048	0.309	D73S	-1.04	0.292
-0.156	-0.144	0.125	-0.23	L47K	-0.47	0.078
-0.026	-0.382	0.021	-0.379	V104P	-2.7	-0.696
-0.077	0.429	0.062	0.453	R107L	0.56	0.639
-0.765	0.717	0.612	-0.542	G37R	-1.14	-0.593
-0.283	-0.097	0.226	-0.678	G89Q	-1.25	-0.572
0.487	0.154	-0.39	0.433	H90V	0.14	0.46
0.054	0.033	-0.043	0.126	Q14D	-0.01	0.391
-0.06	0.035	0.048	-0.131	N11T	0.01	0.303
0.133	-0.252	-0.106	-0.742	N65E	-0.09	-0.525
0.393	-0.08	-0.315	-0.142	L87D	-0.62	0.128
0.086	-2.027	-0.069	-2.513	V77D	-4.38	-3.011
-0.426	0.502	0.341	-0.118	A96R	0.26	0.295
-0.2	1.607	0.16	-0.252	P102L	2.12	0.615
-0.048	-0.074	0.039	0.134	N99R	0.6	0.444
0.483	0.213	-0.387	-0.042	R56V	0.64	0.235
-0.144	0.412	0.115	-0.215	P72V	0.69	0.166
-0.156	-0.108	0.125	-0.273	I31L	-0.78	-0.128

0.36	-0.415	-0.288	-0.437	Q27A	1.02	0.148
0.141	0.074	-0.113	-0.129	D91F	0.01	-0.101
0.034	-0.439	-0.028	-0.725	G38Q	-1.24	-0.747
-0.071	-0.033	0.057	-0.17	D19Q	-0.18	0.164
-0.065	-0.463	0.052	-0.435	G34E	-2.35	-0.55
1.689	-3.385	-1.351	-3.319	F63T	-2.42	-3.432
0.112	0.054	-0.09	-1.07	I24L	0.28	-0.644
-0.084	0.008	0.067	-0.846	N9Q	0.09	-0.429
0.401	-0.874	-0.321	-1.124	A60G	-1.79	-1.244
0.048	0.252	-0.038	-0.405	S21Q	1.24	0.278
-0.7	0.767	0.56	-0.913	G88F	-0.07	-0.81
0.329	-0.517	-0.264	-0.27	Q45A	1.17	0.126
-0.016	-0.254	0.013	-0.254	E61S	-2.47	-0.441
0.536	-0.118	-0.429	-0.334	L36Q	-1.05	-0.308
-0.066	-0.105	0.053	0.156	H25Q	0.25	0.364
0.128	-0.036	-0.102	-0.996	L15I	0.86	-0.63
0.737	-2.546	-0.59	-2.865	I66D	-2.44	-3.139
0.554	0.023	-0.443	-0.078	Q35V	1.2	0.4
0.027	-0.146	-0.021	-0.837	A8E	0.42	-0.29
-0.713	0.623	0.57	-1.261	A23K	-1.29	-1.115
-0.158	0.34	0.127	0.189	D108N	0.06	0.426
-0.033	0.279	0.027	0.103	S12D	0.82	0.731
0.638	-0.125	-0.51	-0.798	R2T	-0.42	-0.627
-0.348	0.858	0.278	0.325	D81R	-0.13	0.528
-0.577	1.049	0.462	-0.386	G53V	0.29	-0.001
-0.282	-0.354	0.226	-0.963	V29P	-3.81	-1.545
0.047	-0.037	-0.038	-0.299	G18V	0.6	0.247
0.392	-0.251	-0.314	-0.982	L5V	0.87	-0.386

-0.096	-0.005	0.077	-0.338	T16A	-0.2	-0.247
-0.104	0.158	0.083	-0.409	A26L	-0.68	-0.176
0.187	-2.405	-0.149	-2.525	V82T	-2.61	-2.741
0.071	0.302	-0.057	-0.964	E64K	-0.46	-0.709
-0.065	0.531	0.052	-0.427	P17Q	1.61	0.277
-0.675	0.579	0.54	-0.602	N95Y	0.19	-0.507
-0.583	0.118	0.466	-0.731	G103D	-1.46	-0.581
-0.136	0.065	0.108	-0.369	I52V	-0.09	-0.069
0.19	0.167	-0.152	-0.714	D84G	1.49	-0.063
-0.258	0.454	0.206	-0.403	I51M	-0.76	-0.422
1.075	-1.103	-0.86	-1.544	Y13P	-0.65	-1.523
1.058	-3.308	-0.846	-2.928	L10G	-3.01	-3.441
-0.546	0.746	0.436	-0.331	F98M	-0.14	-0.309
-0.291	0.27	0.233	0.117	R105A	0.09	0.221
-0.443	0.532	0.354	-0.895	A49F	0.02	-0.765
-0.182	0.119	0.145	-0.509	L39G	-0.07	-0.197
-0.202	0.454	0.162	-0.679	V70Y	-1	-0.754
